# ΔNp63 transcriptionally regulates ATM to control p53 Serine-15 phosphorylation

**DOI:** 10.1186/1476-4598-9-195

**Published:** 2010-07-21

**Authors:** Ashley L Craig, Jitka Holcakova, Lee E Finlan, Marta Nekulova, Roman Hrstka, Nuri Gueven, James DiRenzo, Graeme Smith, Ted R Hupp, Borivoj Vojtesek

**Affiliations:** 1 Cell Signalling Unit, Cancer Research Center, Crewe Road South, Western General Hospital, University of Edinburgh, Edinburgh EH4 2XR, UK; 2 Masaryk Memorial Cancer Institute, Zluty kopec 7, 656 53 Brno, Czech Republic; 3 Queensland Institute of Medical Research, Brisbane QLD 4029, Australia; 4 Department of Pharmacology and Toxicology, Dartmouth Medical School, 7650 Remsen, Hanover NH 03755, USA; 5 KuDOS Pharmaceuticals Limited, 327 Cambridge Science Park, Milton Road, Cambridge CB4 0WG, UK

## Abstract

**Background:**

ΔNp63α is an epithelial progenitor cell marker that maintains epidermal stem cell self-renewal capacity. Previous studies revealed that UV-damage induced p53 phosphorylation is confined to ΔNp63α-positive cells in the basal layer of human epithelium.

**Results:**

We now report that phosphorylation of the p53 tumour suppressor is positively regulated by ΔNp63α in immortalised human keratinocytes. ΔNp63α depletion by RNAi reduces steady-state ATM mRNA and protein levels, and attenuates p53 Serine-15 phosphorylation. Conversely, ectopic expression of ΔNp63α in p63-null tumour cells stimulates ATM transcription and p53 Serine-15 phosphorylation. We show that ATM is a direct ΔNp63α transcriptional target and that the ΔNp63α response element localizes to the ATM promoter CCAAT sequence. Structure-function analysis revealed that the ΔNp63-specific TA2 transactivation domain mediates ATM transcription in coordination with the DNA binding and SAM domains.

**Conclusions:**

Germline p63 point mutations are associated with a range of ectodermal developmental disorders, and targeted p63 deletion in the skin causes premature ageing. The ΔNp63α-ATM-p53 damage-response pathway may therefore function in epithelial development, carcinogenesis and the ageing processes.

## Background

p63 is the founding member of the p53 protein family, and is required for the development of limbs and epithelial structures in vertebrates [1]. The p63 gene expresses at least 6 common transcripts by utilising two distinct promoters (TA and ΔN) and alternative splicing within the 3' end of mRNA that generates α,β and γ isoforms [2]. TAp63 variants contain a p53-like TA1 transactivation domain. ΔNp63 variants lack a TA1 domain, but instead contain a unique 14 amino acid sequence that contributes to the formation of an alternative TA2 transactivation domain [3]. All p63 variants contain a DNA-binding domain and a tetramerisation domain with homology to p53. However, p63 alpha isoforms encode a C-terminal extension containing a SAM protein interaction domain, a conserved functional element found in a range of developmental proteins [4].

Initial studies identified p63 as a robust biomarker for epithelial progenitor, or stem, cells [5]. However, the development of TA- and ΔN-isotype specific reagents revealed that ΔNp63 expression is confined to the basal layer of stratified squamous epithelium, whereas TAp63 variants predominate in suprabasal layers [6]. Similarly, *in vitro *keratinocyte differentiation induces hypoexpression of the predominant ΔNp63α isoform [7]. TAp63 isotypes can transcriptionally activate a subset of p53 target genes involved in cell cycle checkpoint control and apoptosis [8,9]. In contrast, initial reports suggested that ΔNp63 variants had no intrinsic transcriptional activity, but could antagonise TAp63- and p53-dependent target gene transcription [2]. However, recent microarray-based screening approaches have identified the transcriptional targets of distinct p63 isotypes in tumour cells and in immortalised keratinocytes [10]. These studies have revealed that ΔNp63α can either activate or repress the transcription of many target genes involved in multiple cellular processes. The challenge now is to dissect how specific validated ΔNp63α transcriptional targets mediate ΔNp63α physiological function. For instance, loss of ΔNp63α-dependent transcriptional repression of S100A2, p21^WAF1 ^and 14-3-3 correlates with ΔNp63α downregulation during keratinocyte differentiation [7,11].

Our previous studies revealed that UV damage-induced p53 phosphorylation is restricted to the ΔNp63α-positive basal epidermal layer of UV-damaged human skin [12], which provided an opportunity to identify novel physiological regulators of the p53 damage response. Site-specific p53 phosphorylation has already been established to play an important role in regulating the p53 response to UV-damage. For example, p53 mutation at the conserved UV-inducible CK2-site sensitizes mice to UV-induced skin cancer and attenuates the p53 transcription programme in MEFs [13]. In this study we show that a positive association between UV-induced p53 phosphorylation in ΔNp63α-positive immortalised keratinocytes is explained by ΔNp63α-dependent transcriptional control of the ATM kinase.

## Results

### The ATM kinase mediates p53 Serine-15 phosphorylation in immortalised keratinocytes

**Figure 1:**
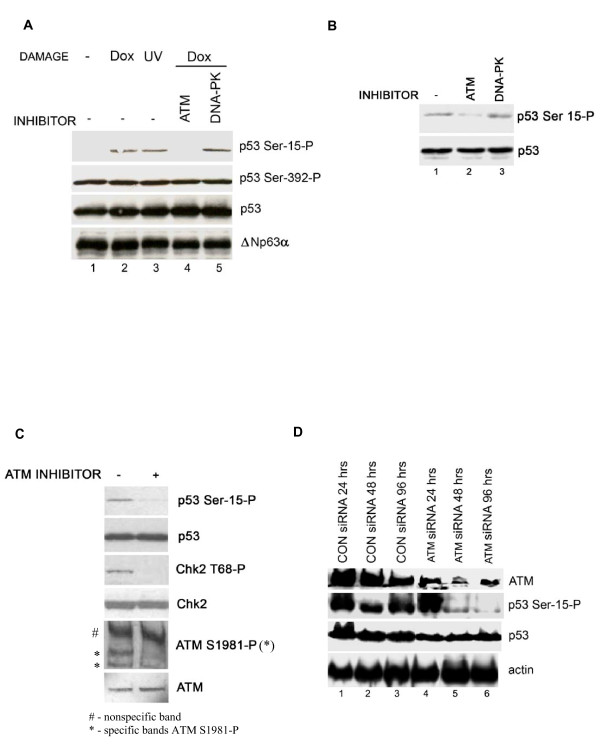
**The ATM pathway is active in HaCaT cells**. (A) Damage-induced p53 Serine-15 is mediated by ATM. HaCat cells were treated with DMSO carrier, 10 μM KU-55933 (ATM inhibitor) or 1 μM NU7441 (DNA-PK inhibitor) for 2 hours, then with 0.5 μM doxorubicin, and cells were harvested after 2hrs, or cells were treated with 20Jm^2 ^UV radiation and harvested after 6hrs. Lysates were blotted for phosphoSerine-15 p53, phosphoSerine-392 p53, total p53 protein and p63, as indicated. (B) ATM inhibition blocks constitutive p53 Serine-15 phosphorylation. Cells were treated with DMSO carrier, 10 μM KU-55933 (ATM inhibitor) or 1 μM NU7441 (DNA-PK inhibitor) for 24 hrs, and lysates were immunoblotted for phosphoSerine-15 p53 and total p53 protein. (C) ATM inhibition blocks constitutive ATM-dependent phosphorylation. Cells were treated with DMSO carrier or 10 μM KU-55933 for 24 hrs, and lysates were immunoblotted for: phosphoSerine-15 p53, phosphoSerine-392 p53, total p53 protein, phosphoThreonine-68 Chk2, total Chk2 protein, phosphoSerine-1891 ATM, and total ATM protein. (D) siRNA-mediated ATM depletion blocks p53 Serine-15 phosphorylation. HaCat cells were treated with control siRNA or siRNA to ATM for 24, 48 or 96hrs, and cell lysates were blotted for ATM, phosphoSerine-15 p53, and total p53 protein.

We previously reported the striking restriction of UV-damage induced p53 site-specific phosphorylation to ΔNp63α-positive epithelial progenitor cells in human skin after UV irradiation *in vivo*. We have now used ΔNp63α-positive/mutant p53 HaCat immortalised keratinocytes [14] as a model system to investigate a potential functional relationship between ΔNp63α and p53 phosphorylation. In this system, basal mutant p53 protein and Serine-392 phosphorylation levels are high and not further stabilised by DNA damage (Figure 1A). In contrast, p53 Serine-15 phosphorylation is low, but is induced following UV-irradiation or treatment with the ATM pathway activator, doxorubicin [15] (Figure 1A, lanes 2 and 3 vs. lane 1). Doxorubicin-induced Serine-15 phosphorylation is attenuated by the small molecule ATM inhibitor, KU-55933 [16] (Figure 1A, lane 2 vs. lane 4), but is unaffected by DNA-PK inhibition. These data confirm that damage-activated ATM signaling functions normally in ΔNp63α-positive HaCat cells. Following increased protein loading or exposure times, basal p53 Serine-15 phosphorylation was easily detectable and was also sensitive to ATM inhibition (Figure 1B, lane 2 vs. lane 1) and insensitive to DNA-PK inhibition. Other key ATM signaling pathway components, including Chk2 Threonine-68 and ATM Serine-1981, are also constitutively phosphorylated (Figure 1C). Treatment with ATM siRNA attenuated both ATM protein expression and p53 Serine-15 phosphorylation (Figure 1D), without affecting p53 levels in comparison with control siRNA. These data confirm that the ATM signaling pathway is primed for damage-activation in ΔNp63α-positive HaCat cells, providing a suitable model system to investigate a potential relationship between ΔNp63α and ATM-dependent p53 phosphorylation.

### ΔNp63α controls ATM expression and ATM-dependent phosphorylation

**Figure 2:**
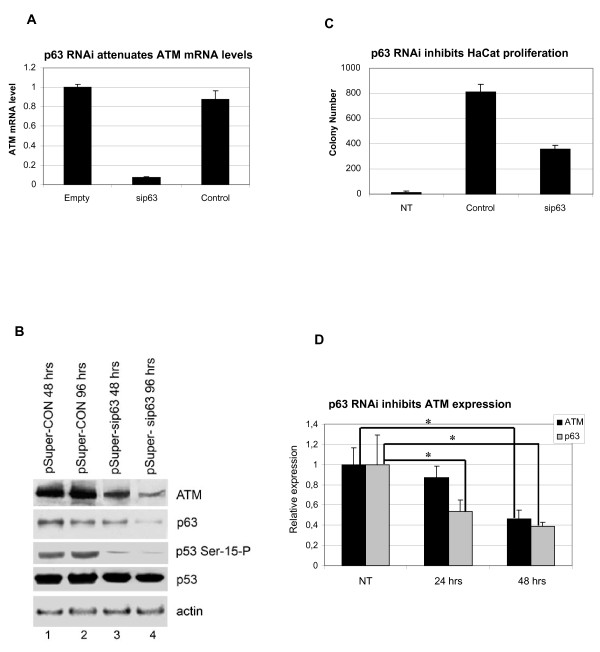
**p63 depletion attenuates ATM expression and ATM-dependent phosphorylation**. (A) pSUPER-p63 RNAi attenuates ATM mRNA levels. HaCat cells were transfected with 1 μg pSUPER-CON or pSUPER-p63si vectors, and selected for geneticin resistance for 4 days. mRNA was extracted from surviving cells and real-time RT-PCR was used to quantitate changes in ATM mRNA expression. Data is represented as fold-change over empty vector control. (B) pSUPER-p63si inhibits ATM expression and p53 Serine-15 phosphorylation in HaCat cells. pSUPER-CON or pSUPER-p63si transfected HaCat cells were harvested after selection for 48 and 96 hours, and immunoblotted for the indicated proteins. (C) pSUPER-p63 RNAi inhibits HaCat cell proliferation. HaCaT cells were transfected with pSUPER-CON or pSUPER-p63si vectors, selected for geneticin resistance for 14 days, and surviving colonies were giemsa stained and counted. (D) siRNA-mediated p63 depletion attenuates ATM mRNA expression. HaCat cells untreated (NT) or treated with control siRNA or p63-targeted siRNA were harvested after 24 hrs or 48hrs and analysed by real-time RT-PCR for p63 and ATM mRNA expression. Data is normalised to β-actin and represented as fold-change over mRNA level in cells treated with control siRNA in particular time point. The data represent the average of three independent experiments + SD. The symbol asterisk denotes significant difference with p values < 0.05 determined by Mann-Whitney test, from the cells treated with control siRNA.

We next used two different methods to inhibit p63 expression, and determined their effects on ATM expression and ATM-dependent basal and damage-induced phosphorylation. Firstly, transfection of HaCat cells with the pSUPER-p63si expression plasmid [17] reduced ΔNp63α mRNA (Figure 2A) and protein expression levels (Figure 2B). Depletion of ΔNp63α protein correlated with reduced ATM protein expression and basal p53 Serine-15 phosphorylation in contrast with transfection of the pSUPER-CON plasmid (Figure 2B). These data suggest that ΔNp63α-mediated control of ATM levels regulates ATM-dependent p53 phosphorylation. Consistent with a role for ΔNp63α in epithelial stem cell maintenance [18], RNAi-mediated p63 depletion reduced colony survival (Figure 2C). Secondly, treatment with p63-directed siRNA oligonucleotides depleted ΔNp63α mRNA levels after 24 hours and ATM mRNA levels were reduced after 48 hours (Figure 2D). These effects on ATM mRNA occurring at later timepoints than downregulation of p63 mRNA is consistent with an ATM regulatory role for ΔNp63α.

### ΔNp63α controls phosphorylation of overexpressed p53

We next used p63 overexpression in the p53-deficient and p63-deficient non-epithelial cell lines to (i) investigate whether ΔNp63α-dependent ATM regulation could be reconstituted and if so, (ii) to perform structure-function analyses to delineate the molecular mechanisms involved.

**Figure 3:**
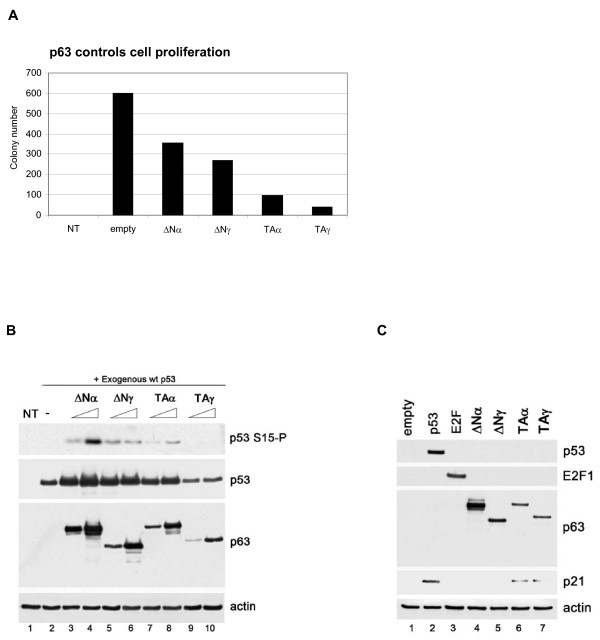
**ΔNp63 isotypes stimulate ATM-dependent phosphorylation**. (A) p63 controls clonogenic cell growth in tumour cells. H1299 cells were transfected with 1 μg p63 plasmids TAα, TAγ, ΔNα, or ΔNγ, or empty vector control, colonies were giemsa stained after 14 days selection and counted. (B) ΔNp63 isoforms stimulate p53 Serine-15 phosphorylation. H1299 cells were cotransfected with 0.2 μg p53 and 0.5 - 1 μg p63 variant as indicated and harvested after 48hrs. Lysates were blotted for the indicated proteins. (C) TAp63 isoforms induce p21^WAF1 ^protein expression. H1299 cells were transfected with 1 μg p53, E2F-1, p63 isoforms TAα, TAγ, ΔNα, or ΔNγ, or empty vector control, and harvested after 48 hours. Lysates were immunoblotted for the indicated proteins.

In line with previous reports, overexpression of p63 variants TAp63α, TAp63γ, ΔNp63α and ΔNp63γ was growth inhibitory (Figure 3A) [19]. TAp63 α and γ overexpression strongly suppressed colony formation (Figure 3A) and correlated with expression of the p53 target and CDK inhibitor, p21^WAF1 ^(Figure 3C), whilst ΔNp63 isoforms did not induce p21^WAF1 ^and only weakly suppressed cell growth. Coexpression of ΔNp63α and ΔNp63γ with p53 specifically increased p53 Serine-15 phosphorylation and expression of the TAp63 isoforms had very weak or no effect on p53 phosphorylation (Figure 3B). Similarly, basal Threonine-68 phosphorylation of exogenous Chk2 was high, but was also specifically stimulated by ΔNp63α coexpression (data not shown). The presence of multiple p63 bands is likely due to cleavage of the full-length isoforms. As reported previously, we noted that TAp63γ protein is less stable than other p63 isoforms, (Figures 3B, C) [2,20].

### ΔNp63 isotypes stimulate the ATM promoter

**Figure 4:**
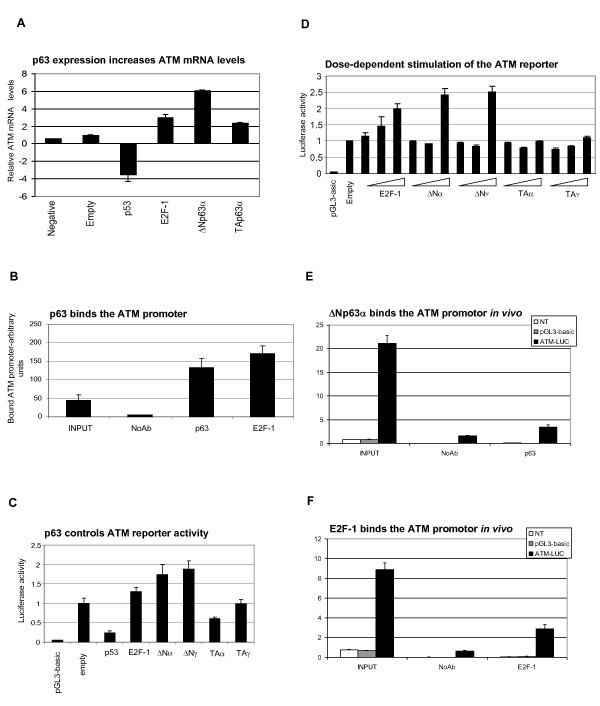
**ΔNp63 isotypes bind the ATM promoter in vivo and stimulate ATM transcription**. (A) The indicated genes (1 μg) were transfected into Saos2 cells. Cells were harvested after 24 hrs, total RNA was isolated and analyzed for ATM gene expression by real time RT-PCR. ATM gene expression was normalized to β-actin, and the data is represented as fold-change over empty vector control. (B) ΔNp63α and E2F-1 bind the endogenous ATM promoter in vivo. Protein-DNA complexes in cycling HaCat cells were crosslinked and analysed by chromatin immunoprecipitation using anti-p63 or anti-E2F-1 antibodies or no antibody control. Crosslinks were reversed and purified DNA was quantified by real-time PCR using ATM primers. Relative amounts of INPUT DNA were 1/10 for ATM. (C) ΔNp63 isoforms stimulate ATM -LUC reporter activity. H1299 were co-transfected with 1 μg ATM-LUC, 0.2 μg pRL-CMV plasmids, and 1 μg empty pCDNA3.1, p53, E2F1 plasmids, or p63 plasmids expressing ΔNα, ΔNγ, TAα or TAγ isoforms. Cells were harvested after 24 hrs and luciferase activity was analysed by Dual-Luciferase reporter assay kit (Promega). Data is normalised to the empty vector control. (D) The ATM promoter is more sensitive to E2F-1 than ΔNp63α. H1299 cells were transfected with 10ng, 100ng or 1000ng E2F-1 or p63 expression plasmids, and reporter plasmids, and harvested as described above. Specific ATM reporter activity was determined as described previously (C). (E)-(F) ΔNp63α and E2F-1 bind an exogenous ATM promoter *in vivo*. H1299 cells were transfected the ATM-LUC reporter plasmid, and co-transfected with ΔNp63α (E) or E2F-1 (F) expression plasmids Protein-DNA complexes were crosslinked after 24 hrs, and ChIP analysis was done using anti-p63 or anti-E2F-1 antibodies. Crosslinks were reversed and purified DNA was quantified by real-time PCR using ATM primers. Relative amounts of INPUT DNA were 1/100.

ΔNp63α-mediated regulation of the ATM pathway may involve effects on either ATM mRNA expression or protein stability. To address the former possibility, we next investigated whether ΔNp63α overexpression affected ATM steady-state mRNA levels. Basal ATM transcription was detectable in cells transfected with control vector (Figure 4A). However, ΔNp63α transfection stimulated a 6-fold increase in steady-state ATM mRNA levels, approximately twice the level of stimulation by the established ATM regulator, E2F-1 [21]. We also show that endogenous ΔNp63α binds the ATM promoter *in vivo *(Figure 4B). In contrast, TAp63α function was strongly attenuated compared to that of ΔNp63α, and p53 expression strongly inhibited ATM mRNA expression. Similar data were obtained when the effects of p63 overexpression on an exogenous ATM promoter were measured (Figure 4C). Basal ATM reporter activity was detectable in cells transfected with a control vector. However, this was stimulated approximately 1.8-fold following cotransfection with ΔNp63 α and γ variants, 1.3-fold by the ATM regulator E2F-1, but not stimulated by TAp63α or p53 coexpression. These data indicate that p63-dependent changes in steady-state ATM mRNA expression are primarily due to promoter regulation, and not due to effects on ATM mRNA stability. Titration of p63 or E2F-1 revealed that the ATM promoter was similarly sensitive to ΔNp63 isoforms and E2F-1 (Figure 4D), although maximal levels of ΔNp63-stimulated ATM promoter activity were reproducibly higher than E2F-1-stimulated activity. Relative levels of ΔNp63α and E2F-1 bound to reporter DNA were similar (Figures 4E, F). Thus, differences in ATM promoter stimulation are not likely to reflect differences in p63 and E2F-1 expression in this system. In summary, (i) ΔNp63α binds the ATM promoter and stimulates ATM transcription, and (ii) this in part maintains ATM-dependent phosphorylation of p53.

### The CCAAT element mediates ΔNp63α-dependent stimulation of the ATM promoter

**Figure 5:**
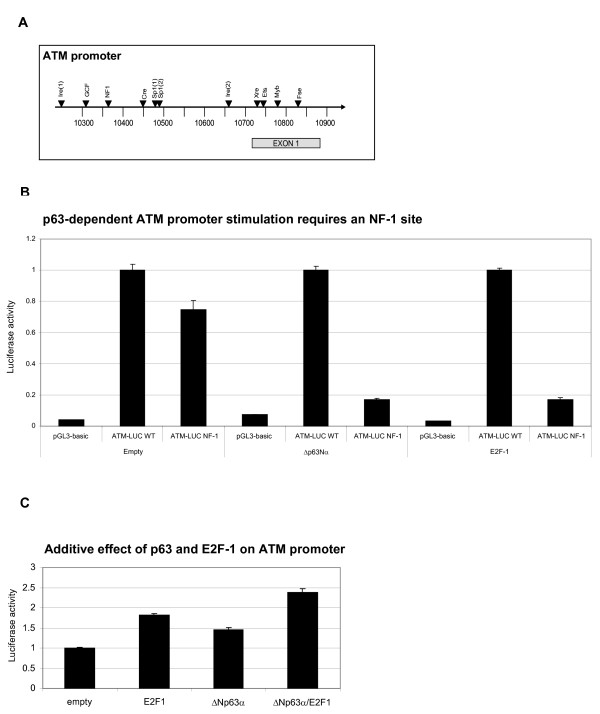
**The CCAAT element mediates ΔNp63α- and E2F-1-dependent stimulation of the ATM promoter**. (A) Schematic representation of the human ATM promoter organisation (Genebank U82828), showing the position of putative response elements which are mutated in this study. (B) The CCAAT sequence mediates ΔNp63α and E2F-1 stimulated ATM transcription. H1299 cells were co-transfected with 1 μg ΔNp63α respective with 1 μg E2F-1, 1 μg ATM-LUC (wt or CCAAT mutant) and 0.2 μg pRL-CMV control plasmids, then lysed and processed after 24 hrs. Specific ATM-LUC activity was determined as described in Figure 4 throughout, and data is normalised to wild-type reporter activity. (C) ΔNp63α and E2F-1 have additive effects on ATM promoter stimulation. H1299 cells were transfected with 0.5 μg ΔNp63α, E2F-1 or 0.5 μg each plasmid along with 1 μg ATM-Luc (wt or mutant) and 0.2 μg pRL-CMV control plasmids, and cells were harvested and processed after 24 hrs. Total DNA was balanced with empty pCDNA3.1 vector.

The DNA binding (DB) domain of p63 is 65% homologous to that of p53, and p63 variants can recognise p53 response elements (REs) [2,22]. Thus, ΔNp63α-mediated ATM transcription could either involve direct binding to the ATM promoter via the DB domain, or indirect binding via an unidentified cofactor. Previous analysis of the ATM promoter failed to identify a p53 RE, although binding sites for several other transcription factors were identified [23]. We therefore used a series of ATM reporter constructs containing point mutations at putative REs to identify sequences required for basal and ΔNp63α-stimulated ATM transcription (see Figure 5A) [23]. Basal ATM reporter activity is absent in mutant ATM reporter constructs lacking either the Ire2 or Fse REs, which were previously found to control ATM transcription in cycling normal human fibroblast and lymphoblastoid cells [23]. ΔNp63α-induced ATM reporter activity was specifically attenuated by mutation of the NF-1 RE, encoded by the AGCCAAT sequence (Figure 5B), and containing a CCAAT element. Mutation of the CCAAT element also blocked E2F-1-mediated ATM promoter stimulation (Figure 5B). This was unexpected as deletion mutagenesis had previously located the E2F-1 target region to between -436 and -392 (Figure 5A 10379-10423), containing two putative E2F-1 REs. However, as this deletion also removes the CCAAT element at -435 - -429 (10380-10386), we reinterpret the original data to indicate that E2F-1 regulates ATM transcription through interaction with the same CCAAT element as ΔNp63α. In support of this, we found that ΔNp63α and E2F-1 do not synergistically activate the ATM promoter, but instead have additive effects (Figure 5C).

### Cooperation of distinct functional domains controls ΔNp63α function

**Figure 6:**
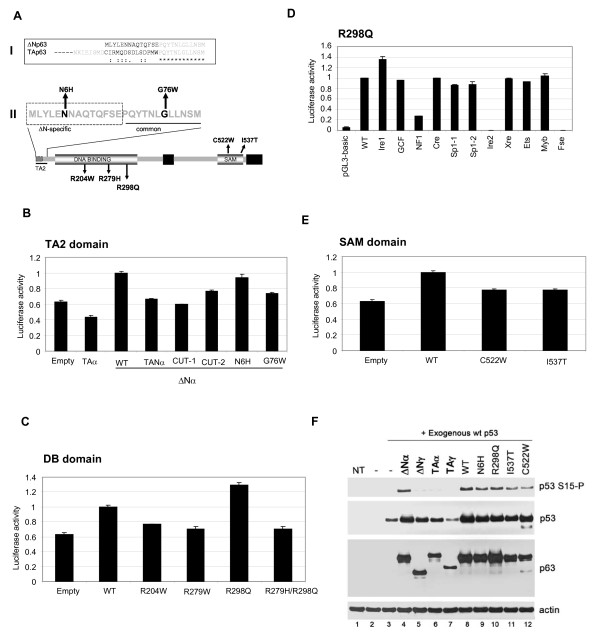
**ΔNp63α mutants alter ATM transcription and ATM-dependent phosphorylation**. (A) (I) Sequence alignment of the amino-termini of TAp63 and ΔNp63, indicating conserved residues. (II) Domain structure of ΔNp63α, indicating sites of point mutations used in this study, and an expansion of the TA2 transactivation domain showing ΔN-specific residues deleted in the CUT-1 mutant, and residues common to both ΔN and TA isoforms that are deleted in the CUT-2 mutant. (B) Effect of TA2 transactivation domain mutation on ΔNp63α-stimulated ATM promoter activity. H1299 cells were co-transfected with 1 μg either wild-type or the indicated mutant ΔNp63α plasmids, and 1 μg ATM-LUC and 0.2 μg pRL-CMV reporter plasmids. Cells were lysed and processed after 24 hrs, and specific ATM reporter activity was determined as in Figure 4 and is normalised to wild-type values. (C) DNA binding domain mutants have opposing effects on ΔNp63α-stimulated ATM promoter activity. H1299 cells were co-transfected with 1 μg either wild-type or the indicated mutant ΔNp63α plasmids, and 1 μg ATM-LUC and 0.2 μg pRL-CMV reporter plasmids. Cells were lysed and processed after 24 hrs, and specific ATM reporter activity was determined as in Figure 4 and is normalised to wild-type values. (D) Mutation of the CCAAT sequence blocks R298Q ΔNp63α-mediated stimulation of ATM transcription. H1299 cells were co-transfected with 1 μg R298Q ΔNp63α, 1 μg ATM-Luc (wt or mutant) and 0.2 μg pRL-CMV control plasmids, then lysed and processed after 24 hrs. Specific ATM reporter activity was determined as in Figure 4 and is normalised to wild-type values. (E) SAM domain mutants attenuate ΔNp63α-stimulated ATM promoter activity. H1299 cells were co-transfected with 1 μg either wild-type or the indicated mutant ΔNp63α plasmids, and 1 μg ATM-LUC and 0.2 μg pRL-CMV reporter plasmids. Cells were lysed and processed after 24 hrs, and specific ATM reporter activity was determined as in Figure 4 and is normalised to wild-type values. (F) Hay-Wells/AEC syndrome-associated ΔNp63α mutants inhibit ATM-dependent p53 Serine-15 phosphorylation. H1299 cells were cotransfected with 0.2 μg p53 and 1 μg of either wild-type or mutant ΔNp63α as indicated, and harvested after 48hrs. Lysates were blotted with phosphoSerine-15 p53, total p53 and p63 protein.

Causative p63 germline mutations have been identified in distinct human developmental disorders characterised by limb deformities and facial clefting. To identify ΔNp63α functional domain(s) involved in ATM regulation, we assayed the effects of both disease-related ΔNp63α point mutants (Table 1) and synthetic deletions on ATM promoter stimulation (illustrated in Figure 6AII).

**Table 1:** Germline p63 point mutations

Point Mutation	Domain	Syndrome
N6H	TA2	ADULT
G76W	TA2	LMS
R204W	DBD "175"	EEC
R279H	DBD "248"	EEC
R298Q	DBD (→TA2*)	ADULT
C522W	SAM	AEC
I537T	SAM	AEC

The functional domain containing each mutation site, and the developmental disorder associated with each mutant is indicated. Numbers in inverted commas ("") refer to the site of homologous hotspot mutations in p53, and TA2* refers to the activation of TA2 function by the R298Q mutation. ADULT, acro-dermato-ungual-lacrimal-tooth; LMS, limb-mammary syndrome; EEC, ectrodactyly-ectodermal dysplasia-clefting; AEC, ankyloblepharon-ectodermal dysplasia-clefting.

### TA2 transactivation domain

The TA2 transactivation domain comprises 14 amino acids unique to the ΔNp63 isoforms and 12 amino acids common to both TA and ΔN isoforms (Figure 6AI). Deletion of the ΔN-specific 14 amino acids blocked ΔNp63α-dependent ATM promoter stimulation (Figure 6B, CUT-1), and replacing the ΔN-specific 14 amino acids with the equivalent TA-specific residues failed to restore ATM transcriptional activity (Figure 6B, TANp63α). Thus, the ΔNp63-specific nucleotides within TA2 are critical for ATM transcriptional control. However, the acro-dermato-ungual-lacrimal-tooth (ADULT) syndrome N6 H mutation within this sequence did not affect ΔNp63α activity, suggesting that the clinical phenotypes of this disease are not mediated by loss of ATM function. In contrast, deletion of the 12 common TA2 domain residues reduced ATM promoter stimulation by roughly 50% (Figure 6B, CUT-2). The limb-mammary syndrome (LMS)-related G76W mutant located within this sequence had a similarly reduced ATM transactivation potential, indicating that Glycine-76 makes a critical contribution to ΔN-specific p63 transcriptional activity (Figure 6B, G76W), and that reduced TA2-dependent transcription may be a causative factor in this disease.

### DB domain

Several p63 DB domain mutants associated with Ectrodactyly-ectodermal dysplasia-cleft syndrome (EEC) syndrome are homologous to p53 tumour-associated hotpoint mutants that disrupt p53 DNA-binding function. The ΔNp63α R204W (equivalent to p53 R175) and R279 H (equivalent to p53 R248H) mutants had a reduced ability to stimulate the ATM promoter (Figure 6C), implicating the DB domain in ΔNp63α-mediated ATM transcription. Interestingly, the ADULT syndrome R298Q DNA-binding domain mutant, previously reported to enhance ΔNp63γ TA2-dependent transcription through an intramolecular mechanism [3], stimulated the ATM promoter 1.3-fold relative to wild-type ΔNp63α (Figure 6C). We showed that hyperactivity of this mutant was mediated solely by the ATM promoter CCAAT element (Figure 6D) and that it was ablated in a double site R298Q/R279 H mutant (Figure 6C).

### SAM domain

Although both α and γ ΔNp63 isotypes stimulated an exogenous ATM promoter to similar levels, only ΔNp63α effectively induced intrinsic ATM kinase activity, suggesting that C-terminal p63 domains specific to the alpha-isotype are essential to regulate the endogenous ATM promoter. Alpha splice variants of both p63 and p73 contain a SAM protein-protein interaction domain that may be important for cofactor recruitment. The NMR structure of the p63 SAM domain revealed a 5 alpha helix bundle with a hydrophobic core [24], which is disrupted by ankyloblepharon-ectodermal dysplasia-clefting (AEC)-specific point mutations [25]. Ectopic expression of ΔNp63α AEC mutants C522W or I537T had an attenuated ability to stimulate the ATM promoter (Figure 6E), and both mutations blocked ΔNp63α-dependent stimulation of p53 Serine-15 phosphorylation (Figure 6F).

These data indicate critical roles for the TA2, DB and SAM domains in regulating ΔNp63α-dependent ATM transcription, and suggest that reduced ATM function may be a factor in the clinical manifestation of specific p63 germline disorders [25].

## Discussion

p53 is the primary mediator responsible for removing DNA damaged epidermal cells [26], and p53 phosphorylation at the CK2-site is required to suppress UV-induced skin cancer development in mice [13]. We previously reported the striking confinement of UV-induced p53 phosphorylation at the key damage-response CK2 and ATM sites to ΔNp63α-positive basal skin cells, despite substantial p53 stabilization throughout the epidermis [27]. We next aimed to identify novel factors that control damage-induced p53 phosphorylation in a keratinocyte model system, and discovered that the epithelial stem cell marker ΔNp63α is a novel ATM regulator that controls p53 Serine-15 phosphorylation through transcription of the ATM kinase. Loss of ΔNp63α by RNAi or differentiation reduced ATM-dependent phosphorylation and conversely, ΔNp63α overexpression stimulated ATM signaling. A recent genome-wide screen reporting that ATM expression is reduced by 30-60% in p63 siRNA-treated epithelial cell lines supports our finding [28].

Post-translational activation of the ATM kinase by ionizing radiation, oxidative stress, chemotherapeutic drugs [15] and UV radiation is well-established [29]. However, ATM transcriptional regulation has been shown to occur both *in vitro *and *in vivo *[30]. E2F-1 stimulation of ATM transcription [21] has been implicated in oncogene-mediated p53 activation [31]. In contrast, epidermal growth factor sensitizes cells to ionizing radiation through Sp1-mediated repression of ATM transcription [32]. We have shown that p63 ΔN-isotypes are novel positive regulators of ATM transcription that interact with the promoter CCAAT sequence. p63-dependent gene regulation has been reported to occur through interaction of the DB domain with a p53 RE [22]. However, the lack of similarity of the CCAAT sequence to classical p53 REs suggests that p63 interaction with a CCAAT element is indirect, and requires a CCAAT-binding mediator. We show that the E2F-1 regulates ATM transcription through the same CCAAT sequence, not a canonical E2F-1 response element, suggesting that a CCAAT-binding cofactor integrates activation signals from diverse ATM transcriptional regulators. CCAAT-binding proteins include NF-1/CTF [33], NF-Y [34] and C/EBP [35]. NF-Y can mediate ΔNp63α-dependent transcription in human keratinocytes [10,36], p53-dependent repression of cell cycle genes [37], and transcriptional activation by p53 gain-of-function mutants [38]. However, we found that coexpression of the NF-YA isoform inhibits ΔNp63α stimulation of the ATM reporter (data not shown), presumably by displacing the unidentified ΔNp63α coactivator from the ATM promoter CCAAT element. Ongoing studies aim to further delineate the mechanism of ΔNp63α-mediated ATM transcriptional control by identifying ΔNp63α binding partners in epithelial cells.

Based on our findings so far, cooperation of three distinct functional domains is required to mediate p63-dependent ATM transcription. We found that p63 ΔN-isotypes transcriptionally activate the ATM gene, whereas TA-isotypes do not, highlighting an essential role for the TA2 transactivation domain in mediating ΔNp63α function. Future studies will aim to determine which cofactors are recruited to this region, and whether their access is controlled by TA2 domain post-translational modification, similar to the p53 model [39]. There was also a requirement for an intact p63 DB domain, despite the absence of a canonical p53 RE in the ATM promoter. However, in addition to providing a surface for the sequence-specific binding of DNA, the p53 DB domain modulates p53 function by providing a contact interface for regulatory proteins such as ASPP1, Mdm2, and DAPK superfamily kinases [40,41], and the high degree of conservation of the p63 DB domain suggests that a similar interface exists on p63. Finally, the p63 SAM domain forms a binding site for NF-Y [36], and SAM domain disease-associated mutants have decreased transcriptional repressor and activator function [7,42]. We found that AEC point mutations within the SAM domain [25] inhibited ΔNp63α-stimulated ATM transcription and ATM-dependent p53 phosphorylation, indicating that this domain may be essential for cofactor recruitment by the ΔNp63α. Interestingly, the AEC clinical phenotype predominantly involves skin defects without associated limb abnormalities [42], consistent with a skin-specific role for ΔNp63α-ATM-p53 signaling in mediating normal ectodermal development. Therefore, the coordinated assembly of several cofactors may be required for fully functional p63 transcriptional machinery.

According to our model, elevated ΔNp63α-dependent ATM transcription primes p53 leading to damage-sensitivity in epithelial stem cells. Loss of p63-ATM-p53 pathway function will compromise epithelial stem cell function and promote premature ageing or skin carcinogenesis. Interestingly, transgenic mice with a specific p63-deficiency in the epithelium show increased senescence and an accelerated ageing phenotype [43]. Although transgenic mice lacking the Serine-18 (equivalent to human Serine-15) ATM phosphorylation site are not cancer-prone [44], it is now important to determine whether mutation at p53 Serine-18 enhances sensitivity to UV-induced skin tumorigenesis, similar to mutation of the CK2-site. Interestingly, p53S18A/S23A (ATM-/CHK2-sites) double mutant mice develop a spectrum of spontaneous tumours distinct from p53S23A and p53-null mice, and show accelerated skin ageing phenotypes when crossed into a repair-deficient background [45].

Further, activation of the ATM-CHK2 pathway during early tumorigenesis has been reported to provide a selective pressure for p53 mutation [46]. The discovery that the ΔNp63 promoter is subject to both p53-mediated activation and repression by ΔNp63α [17], and that ATM-dependent phosphorylation mediates ΔNp63α degradation [47] suggests that activity of the damage-response ΔNp63α-ATM-p53 pathway is finely modulated by complex feedback mechanisms. Further dissection of this pathway should provide molecular targets for combating cancer and ageing.

## Materials and methods

### Cell Treatments

HaCat and Saos-2 cells were maintained in DMEM supplemented with 10% FCS. H1299 cells were maintained in RPMI supplemented with 10% FCS. p63 expression plasmids were obtained from Dr Karin Nylander, and transient transfections were done using lipofectamine LF2000 (Invitrogen). Ambion silencerTM siRNA oligonucleotides were used to block ATM expression: sense 5'-gccagcaaauucuagugcctt -3' antisense: 5'-ggcacuagaauuugcuggctc-3'. Transfection of HaCaT cells with 200 pMol ATM siRNA used the siPORTTM NeoFXTM transfection reagent. Dharmacon ON-TARGETplus SMARTpool p63 siRNA was used to knockdown p63 expression using the DharmaFECT 1 transfection reagent. ON-TARGETplus siCONTROL Non-targeting pool was used for control transfections. pSUPER-p63si stable transfections were done as previously described [17].

### Site-directed mutagenesis

ΔNp63α site-directed mutagenesis used the QuikChange^® ^Site-Directed Mutagenesis Kit (Stratagene) and the following primers:

N6H-For 5'-TTGTGAAATGGTGCCCTAACCATGAGCTGAGCCGTG-3'; N6H-Rev 5'-AATTGAGTCTGGGCATTGTGTTCCAGGTACAAC-3'; G76W-For 5'-GTACACGAACCTGTGGCTCCTGAACAGCATGG-3'; G76W-Rev 5'-CCATGCTGTTCAGGAGCCACAGGTTCGTGTAC-3'; R204W-For 5'-TTGTGAAATGGTGCCCTAACCATGAGCTGAGCCGTG-3'; R204W-Rev 5'-CACGGCTCAGCTCATGGTTAGGGCACCATTTCACAA-3'; R279H-For 5'-GCTGCGTCGGAGGAATGAACCATCGTCCAATTTTAATC-3'; R279H-Rev 5'-GATTAAAATTGGACGATGGTTCATTCCTCCGACGCAGC-3'; R298Q-For 5'-CAAGTCCTGGGCCAACGCTGCTTTG-3'; R298Q-Rev 5'-CAAAGCAGCGTTGGCCCAGGACTTG-3'; C522W-For 5'-GTTGGGCTGTTCATCATGGCTGGACTATTTCACGAC-3' C522W-Rev 5'-GTCGTGAAATAGTCCAGCCATGATGAACAGCCCAAC-3'; I537T-For 5'-GACCACCATCTATCAGACTGAGCATTACTCCATG-3'; I537T-Rev 5'-CATGGAGTAATGCTCAGTCTGATAGATGGTGGTC-3'; CUT1-For 5'-GGCCTCGAGCCACAGTACACGAACCT-3'; CUT1-Rev 5'-ACCTCTAGATCATTCTCCTTCC-3'; CUT2-For1 5'-GGCCTCGAGGACCAGCAGATTCAGAAC-3'; CUT2-For2 5'-GGCCTCGAGTTGTACCTGGAAAACAATGCCCAGACTCAATTTAGTGAGGACCAGCAGATTCAGAAC-3'; CUT2-Rev 5'-ACCTCTAGATCATTCTCCTTCC-3'; TAN-For 5'-GGCCTCGAGTGTATCCGCATGCAAGACTCAGACCTCAGTGACCCCATGTGGCCACAGTACACGAACCT-3'; TAN-Rev 5'-ACCTCTAGATCATTCTCCTTCC-3'.

### Immunoblotting

Immunoblotting was done essentially as described previously [40]. p53 protein was detected using DO-1 and DO-12 anti-p53 antibodies, specific p53 Serine-15 phosphorylation was detected using p53 phosphoSerine-15 antibodies (New England Biolabs), and all p63 isoforms were detected using the 4A4 antibody (Abcam). Anti-ATM (5C2, GeneTex) and anti-ATM phosphoSerine-1981 (clone 10H11.E12, Upstate) antibodies were used.

### Reporter Assays

Wild-type and mutant human ATMpLUC reporter plasmids [23] and the Arf exon1 βpLUC reporter plasmid [48] were previously described. 1 μg of expression plasmid, 1 μg of reporter plasmid and 0.2 μg of pRL-CMV plasmid were cotransfected into H1299 cells using lipofectamine, and cells were harvested after 24 hours. Reporter activity was determined using the Dual-Luciferase reporter assay kit (Promega).

### Colony Formation Assays

H1299 cells were transfected with 1 μg of p63 expression plasmid, and selected using 1 μg/ml geneticin (Invitrogen). After 14 days, colonies were stained with Giemsa and counted.

### Real-Time PCR

Total mRNA was extracted using the Qiagen RNeasy Kit, and 40ng samples were analysed by real-time RT-PCR using Quantitect^® ^SYBR^® ^Green detection. RT-PCR conditions were: 50°C for 30 min, 95°C, 15 min, and 44 cycles of 95°C for 15 sec, 55°C for 30 sec, 72°C for 45 sec. Melting curves were recorded from 60°C to 95°C. Primers were: p63-For 5'-GGAAAACAATGCCCAGACTC-3'; p63-Rev 5'-GCTGTTCCCCTCTACTCGAA-3'; ATM-For 5'-CCAGGCAGGAATCATTCAG-3'; ATM-Rev 5'-CAATCCTTTTAAATAGACGGAAAGAA-3'; Actin-For 5'-CTACGTCGCCCTGGACTTCGAGC-3'; Actin-Rev 5'-GATGGAGCCGCCGATCCACACGG-3'.

### Chromatin Immunoprecipitation Assays

4 μg of 4A4 (Abcam) antibody was used to immunoprecipitate p63-DNA complexes, and 4 μg of KH95 antibody (Santa Cruz) was used to immunoprecipitate E2F-1-DNA complexes. 2-5 μl purified DNA was analysed by real-time PCR, and input DNA dilutions are indicated in figure legends. Primers used were:

ATM For 5'-AAAACCACAGCAGGAACCAC-3'; ATM Rev 5'-TCCAAGTCTGAGGACGGAAG-3'; GAPDH For 5'-AAAAGCGGGGAGAAAGTAGG-3'; GAPDH Rev 5'-CTAGCCTCCCGGGTTTCTCT-3'. The programme used was: 95°C, 15 min, then 40 cycles of 95°C, 15 sec, 56°C, 30 sec, 72°C, 30 sec, and product melting curve was read from 60°C to 95°C at 1°C intervals.

### Reporter ChIP

10 cm culture dish of H1299 cells were transiently transfected with 6.7 μg ΔNp63α or HA-E2F-1 expression plasmids 1.67 μg pGL3-basic or ATMpLUC and 1.67 μg pRL-CMV. Cells were crosslinked after 24 hrs and processed as outlined above.

## Abbreviations

ATM: Ataxia telangiectasia mutated; SAM: Sterile alpha motif; DNA-PK: DNA-dependent protein kinase; MEFs: Mouse embryonic fibroblasts; E2F-1: E2F transcription factor 1; REs: Response elements; NF-1: Nuclear factor 1; ADULT: Acro-dermato-ungual-lacrimal-tooth syndrome; LMS: Llimb-mammary syndrome; EEC: Ectrodactyly-ectodermal dysplasia-cleft syndrome; NMR: Nuclear magnetic resonance; AEC: Ankyloblepharon-ectodermal dysplasia-clefting; CTF: CCAAT box-binding transcription factor; NF-Y: Nuclear factor Y; C/EBP: CCAAT/enhancer binding protein; ASPP1: Apoptosis-stimulating protein of p53; Mdm2: Murine Double Minute 2; DAPK: Death-associated protein kinase;

## Competing interests

The authors declare that they have no competing interests.

## Authors' contributions

Study concept and design: ALC, JH, LE; acquisition of data: ALC, JH, LF, MN, NG, JD, GS; analysis and interpretation of data: ALC, JH, LF, MN, NG, JD, GS; drafting of the manuscript: ALC, JH, LF; critical revision of the manuscript for important intellectual content ALC, TRH, BV; statistical analysis: RH; study supervision: TRH, BV. All authors have read and approved the final manuscript.
